# Precision Medicine in Neonates: A Tailored Approach to Neonatal Brain Injury

**DOI:** 10.3389/fped.2021.634092

**Published:** 2021-05-19

**Authors:** Maria Luisa Tataranno, Daniel C. Vijlbrief, Jeroen Dudink, Manon J. N. L. Benders

**Affiliations:** Department of Neonatology, Wilhelmina Children's Hospital/University Medical Center Utrecht, Utrecht University, Utrecht, Netherlands

**Keywords:** personalized medicine, brain injury, intraventricular hemorrhage, stroke, newborn, preterm, artificial intelligence, precision medicine

## Abstract

Despite advances in neonatal care to prevent neonatal brain injury and neurodevelopmental impairment, predicting long-term outcome in neonates at risk for brain injury remains difficult. Early prognosis is currently based on cranial ultrasound (CUS), MRI, EEG, NIRS, and/or general movements assessed at specific ages, and predicting outcome in an individual (precision medicine) is not yet possible. New algorithms based on large databases and machine learning applied to clinical, neuromonitoring, and neuroimaging data and genetic analysis and assays measuring multiple biomarkers (omics) can fulfill the needs of modern neonatology. A synergy of all these techniques and the use of automatic quantitative analysis might give clinicians the possibility to provide patient-targeted decision-making for individualized diagnosis, therapy, and outcome prediction. This review will first focus on common neonatal neurological diseases, associated risk factors, and most common treatments. After that, we will discuss how precision medicine and machine learning (ML) approaches could change the future of prediction and prognosis in this field.

## Introduction

Despite enormous advances in neonatal care to prevent neonatal brain injury and future neurodevelopmental impairment, predicting long-term outcome in neonates at risk for brain injury remains difficult. Parents and families of newborns admitted to the NICU with brain injury inevitably face many unknowns. In the initial period after birth, their first question will usually be: “Will my baby survive?” immediately followed by other fundamental questions such as: “What kind of future can we expect for our child? And for us as a family?” or “Will my baby be able to walk? Will he/she go to school?”

Currently, prediction of outcome is based on developmental milestones measured at specific ages. Early prognosis is based on CUS, MRI, EEG, and/or general movements assessment (GMA) assessed during follow up visits. These methods' predictive power is based primarily on population data reflecting the general outcome in similar children. Thus, predicting the outcome in a specific individual (personalized prediction medicine) is not yet possible and is urgently needed. Estimating the most accurate prognosis, as early as possible, is essential to adequately inform the child's family and begin intervention therapy even before the onset of clinical symptoms, particularly given that the brain's plasticity is highest in the first few months after birth. An individualized approach to neonatal brain injury and neurologic-oriented precision medicine is warranted for fragile neonates, not only for outcome prediction but also for preventing or reducing neonatal brain injury and supporting decision-making. Perinatal conditions leading to brain injury in the neonatal period include **hypoxia-ischemia, arterial ischemic stroke, and intraventricular hemorrhage** and especially its complications [post-hemorrhagic ventricular dilatation (PHVD) and periventricular venous hemorrhagic infarction (PVHI)], primary causes of neonatal mortality, and life-long disabilities such as epilepsy and cerebral palsy (1, 2). It has become clear that there is a need for individual and precise information on the spectrum of risk factors, symptoms, early detection, type, and location of brain injury to design/initiate effective therapeutic and supportive strategies.

In a NICU, medical professionals are continuously trying to obtain as much information as possible on the patients in their care. Education and experience provide them with the skills to make the right decisions. However, the patient load is high. Furthermore, the human mind can only recall the outcome of the most recent or complicated case. Computer programs can approach human cognitive tasks. Thus, a possible approach to fulfill the needs of modern neonatology is developing new tools for a precision medicine approach based on large databases, and machine learning (ML) applied to neuromonitoring and neuroimaging data and genetic analysis and assays measuring multiple biomarkers (omics). A synergy of all these techniques and the use of automatic quantitative analysis could give clinicians the possibility to provide patient-targeted answers to parents' questions. Artificial intelligence can mimic human experience-based-learning with ML supervised by experts. ML learns from past experiences, identifies trends and patterns in data, and uses it to build a model or algorithm. These algorithms can be used afterwards to make predictions on new data as a supportive-decision making tool. ML models can be created from data where the outcome is known (supervised learning). Also, ML can be used to identify patterns in data without previous knowledge (unsupervised learning).

This review will first focus on common neonatal neurological diseases such as perinatal hypoxia-ischemia, perinatal ischemic stroke and intraventricular hemorrhage and their risk factors and most common treatments. Afterwards, we will focus on how precision medicine and ML approaches might accurately identify infants who will develop HIE and cerebral palsy. We will primarily discuss the newest (and with highest predictive value) clinical, neurophysiological, neuroimaging, and “omics” techniques, that in our opinion, could change the future of prediction and prognosis in this field. However, we are aware that the present review cannot be comprehensive of all the techniques in the field, therefore we chose to focus on a limited list where the first steps are already taken toward a more individualized neonatal care and a better prediction.

### Hypoxic-Ischemic Encephalopathy

Hypoxic ischemic encephalopathy (HIE) is characterized by a disturbed neurologic function in the perinatal period, manifesting with an abnormal level of consciousness, seizures, respiratory insufficiency, and depressed tone and reflexes (3). Currently, the only effective treatment to reduce death or severe long-term neurological impairment is therapeutic hypothermia, which led to an increase in survival rate, with a persistent rate of death and disabilities around 16–30% (4, 5). However, timing of intervention is a significant factor in improving outcome and treatment efficacy (6).

Multiple mechanisms are involved in brain injury pathogenesis, such as hypoxia-ischemia, inflammation, excitotoxicity, and oxidative stress (7). The degree and extent of injury and individual vulnerability depends on sex, genetic background, maturational age, and the extent of brain injury and the degree of brain development of particular regions at the moment of insult (8, 9). Antenatal conditions such as maternal infection/inflammation, intrauterine growth restriction *in utero* hypoxia can also influence and modulate vulnerability to brain injury (7). Furthermore, different stages of brain injury can be recognized, and, for each stage, different mechanisms are involved. This information is critical to program therapeutic interventions (10, 11). Recent findings and ongoing studies, using ML-based on big data and -omics approaches, suggest that by combining clinical, neurophysiological, neuroimaging, and metabolic/(epi)genetic data, it might be possible to identify infants who will develop NE and cerebral palsy accurately, shortly after birth (12, 13). This would allow early initiation of therapy. However, these methods are currently not yet available at the bedside.

### Perinatal Arterial Ischemic Stroke

Perinatal arterial ischemic stroke (PAIS) is a relatively common (birth-prevalence in term and near-term newborns ranges from 6 to 17/100,000) (14) and a severe neurologic disorder affecting primarily term infants (15). The actual treatment is supportive; however, neuroprotective approaches have been developed and are currently under evaluation in clinical trials. Among them, therapeutic hypothermia, erythropoietin, and stem cell therapy showed promising results in pre-clinical and pilot studies (16–18).

Sex (male), obstetrical conditions (first pregnancy, caesarean section), and perinatal complications such as perinatal hypoxia, and foetal/neonatal inflammatory state, are most commonly associated with neonatal stroke (19). In general, most studies emphasize the role of maternal/fetal infection/inflammation (20). Inherited or acquired prothrombotic status contributes minor to the PAIS (21). The cumulative perinatal risk factors increase the incidence dramatically (22). Other conditions, such as bacterial meningitis, hypoglycemia, and congenital heart disease, may also be involved as risk factors in PAIS development (23).

Few studies using ML have attempted to obtain reproducible automatic segmentation of the stroke lesion volumes, mainly in adults (24). A comparative study evaluating different segmentation (simple vs. complex ML) methods shows that high-level ML methods lead to significantly better segmentation results compared to the relatively simple classification methods. However, none of the methods could achieve results in the range of the human observer agreement (24). Thus, more studies are needed in this field since segmentation can help quantify the size and location of injury to test the efficacy of therapies and prognosis.

### Intraventricular Hemorrhage (IVH), Periventricular Hemorrhagic Infarction (PVHI) and Post-hemorrhagic Ventricular Dilatation (PVHD)

Intraventricular hemorrhage (IVH) and its severe complications: PVHI and PVHD, are common conditions after premature birth and are frequently associated with mortality and adverse long-term neurodevelopmental outcome (25). Regarding treatment, IVH prevention bundles such as delayed cord clamping, minimal handling, midline head position, limiting the number of infusions, and frequent multidisciplinary assessments have emerged as essential tools for reducing IVH morbidity (26–29).

Most relevant risk factors are lower gestational age, absent antenatal steroid treatment, low Apgar scores, pneumothorax, early sepsis, inherited thrombophilia, and the use of inotropic drugs during the first days of life (30, 31). However, recently Tortora et al. (32) suggested that the congenital variation in the vascular architecture of subependymal veins might play a role in the pathogenesis of IVH, especially when other risk factors affecting the cerebral circulation occur. Another recent study demonstrated that the presence and expression of specific vascular endothelial growth factor (VEGF) genetic phenotypes were associated with higher incidence rates of IVH in extremely preterm newborns (33). Regarding the possibility to apply ML to diagnose or prevent consequences of IVH in preterm infants early, one study attempted to determine whether ML techniques would be able to identify specific clusters of risk factors with different probability estimates for severe neonatal morbidity (including IVH) in preterm infants, with promising results (34). However, there is still much room for improvement, and further studies on this field are needed before clinicians will be able to use these tools in daily practice. A first, fundamental step can be to build a big publicly available dataset of clinical data, CUS and MRI images, neurophysiological and biochemical/genetic data by which deep ML models can find more generalized features to improve their performance.

## The Role of Precision Medicine for “Brain Oriented Care”

**Figure 1:**
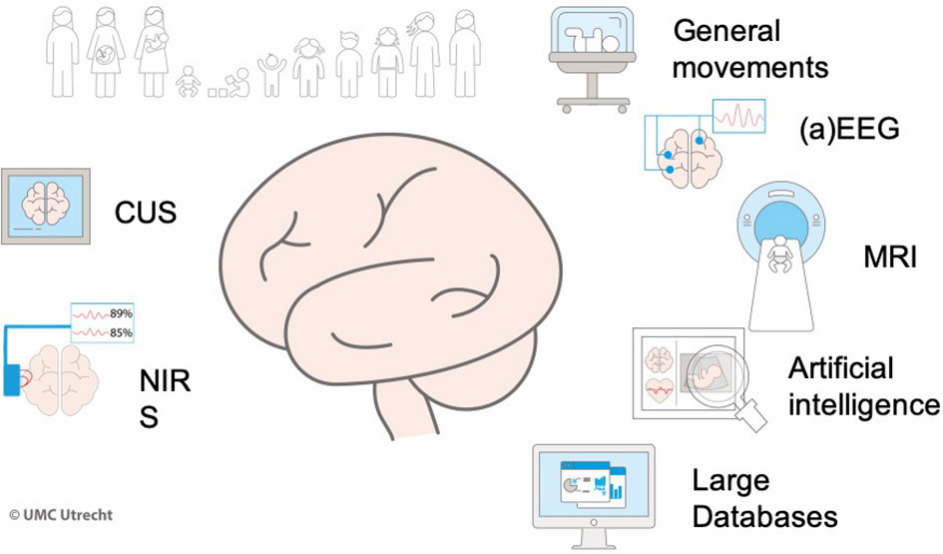
Precision medicine for brain-oriented care.

Advances in neonatal care, specifically “brain oriented care,” particularly the use of therapeutic hypothermia for the treatment of hypoxic-ischemic encephalopathy, paved the way for neuroprotection in newborns at risk for brain injury. A multidisciplinary team for “brain-oriented care” is warranted in the NICU to optimally implement such treatments (26) and provide tailored care. This team should include pediatric neurologists, neonatologists, and “brain-oriented” specialized nurses (26). Furthermore, specific protocols should be combined with neuroimaging (MRI) and neuromonitoring [video multi-channel EEG and amplitude-integrated EEG, near infrared spectroscopy (NIRS)] (Figure 1). Moreover, laboratory support for biomarkers, genetic and metabolic tests, and data scientists to analyze big data providing rapid algorithms for diagnosis and *ad hoc* treatments should be available. Dissemination of knowledge and research personnel and facilities is also warranted. Furthermore, genuinely personalized medicine is unlikely to be realized without the use of artificial intelligence (AI) (35) and ML. In neonatal neurology, ML is used to prevent brain injury from the continuous assessment of vital signs (36). Fairchild et al. (37) used a heart rate characteristic index (HRC index) from the first 28 days after birth in preterm infants and related this to neurodevelopmental outcome. They found an abnormal HRC to correlate with acute brain injury.

Much effort was made to develop automated seizure detection. When tested, all algorithms showed clinically relevant detection rates (38). Doyle et al. (39) and Malarvilli and Mesbah (40) used heart rate variability to detect seizures. Their model showed relevant results with sensitivity and specificity above 80%. Karayiannis et al. (41) designed a trained neural network that was able to distinguish seizures from random infant movements based on video images. In the term infant, encephalopathy severity can be classified based on the EEG signal. Several attempted to grade the degree of abnormality in the EEG of a neonate with hypoxic-ischemic encephalopathy (42). The ML models were suitable as a clinical decision support tool to predict outcome after hypoxic-ischemic encephalopathy (43). Furthermore, evaluating the human connectome and its relation to normal and abnormal development in preterm and term infants is virtually impossible without ML (44). AI and ML are developing fields of research and will be introduced in clinical practice. Moreover, the interpretation of MRI images of term and preterm infants using ML can possibly individualize the outcome prognosis. ML understanding of EEG patterns can potentially guide medical treatment and the use of sedation. EEG and vital parameter analysis can explore sleeping patterns in preterm babies. The potential of automatically warning a caregiver when a baby is sleeping makes true personalized developmental care possible (45, 46).

The combination of clinical and ML tools trained on combined datasets of MRI, EEGs, clinical and biochemical/genetic data would hopefully help clinicians in providing all the treatments and support to mitigate the long-term effects of brain injury through the use of “brain oriented- care” during the whole admission and after discharge in a follow-up program.

Particularly, many efforts have been put into implementing MRI, EEG, GMs, Hammersmith Infant Neurological Examination (HINE) training for clinicians worldwide. However, achieving high education and gain experience in all these techniques is very hard in small centers. Thus, providing a decision support tool derived from ML algorithms can help every physician in decision-making, hopefully, a relevant improvement in long term outcome.

## MRI

The use of magnetic resonance imaging (MRI), a non-invasive neuroimaging method, has revolutionized our knowledge of structural alterations to normal neural development leading to neurological impairment later in childhood (47). MRI can provide detailed info *in vivo* of the fetal and neonatal brain that cannot be obtained in any other imaging modality, helping clinicians define specific risk factors for neonatal brain injury (48).

Standard sequences (T1 and T2-weighted images) provide anatomic detail of the developing brain. They can detect brain injury and lesions linked to common neurological neonatal diseases: hypoxic-ischemic brain injury, perinatal arterial ischemic stroke, IVH-PVHI, infections of the central nervous system, and congenital cerebral malformations. Furthermore, advanced MRI sequences can be used in specific conditions to assess: brain metabolism (MR spectroscopy), the presence of hemosiderin (susceptibility-weighted images), microstructural integrity (diffusion tensor imaging), acute ischemic injury (diffusion-weighted images), cerebral veins and arteries (magnetic resonance angiography and venography), brain perfusion (arterial spin labeling), and function (resting-state functional MRI). Moreover, quantitative approaches can measure brain volumes of all different regions, quantify microstructural integrity and cortical development (47, 48), that are otherwise difficult to quantify by eye. *Ad hoc* protocols and specific methodologies have been developed in order to address the methodological challenges of the newborn population such as: sensitivity to motion, small brain size, different soft-tissue contrast and incomplete maturation of brain structures (47). Thus, the use of neonatal specific MRI post-processing tools is essential in order to obtain reliable results (47).

Recently, newly developed ML techniques have been applied to earlier acquired neonatal MRI databases to predict cognitive scores at 4 years (49). Similar techniques have been used to predict cognitive, and motor development in preterm infants based on the microstructure of white matter regions measured using diffusion tensor imaging (DTI) correlated with the Bayley Scales of Infant-Toddler Development (BSID-III), as well as to predict neurological outcome in patients with neonatal encephalopathy based on connectivity networks (50–54). Moreover, deep learning–methods based on neonatal MRI and brain segmentation analysis have successfully automated classification of impaired brain maturation in full-term infants born with congenital heart disease and have provided insight into the pathogenesis of cerebellar dysplasia (55, 56). However, to optimize and refine the prognostic value of quantitative MRI techniques, it would highly be recommended to use standardized protocols, imaging modalities, and scan timing across centers (57).

### Quantitative MRI Techniques and Outcome Prognosis

Quantitative brain MRI aims to offer objective and reliable measures of brain structure, function, and brain connectivity, in the normal and abnormal brain. The main aims of quantitative neonatal MRI are: the development of automatic algorithms for images interpretation (58), the detection, measurement, and characterization of “subtle” brain abnormalities/injuries (59, 60), and prediction of behavior, cognitive and motor long term outcome based on sophisticated algorithms (61). Quantitative MRI analysis is based on the use of multiple software packages capable of drawing together neuroimaging data processing routines from across, linking them together to implement end-to-end processing and analytic solutions. These solutions not only lead to detailed mathematical and statistical results but also help to improve the reproducibility of measurements and reduce the post-processing duration (62).

Using ML approaches, quantitative analysis of brain morphometry showed significant deviations between different groups of preterm infants (with or without brain injury, extremely/moderately preterm) compared with full-term infants (47). A study comparing visual vs. quantitative MRI assessment of the thalami in infants with HIE showed that both approaches are needed since visual assessment alone can underestimate injury (63).

Automatic methods for brain volume and cortical morphology quantification, early as well as term equivalent age MRI, were good predictive tools of both motor and cognitive outcome at 2–3 years (64, 65). Furthermore, quantitatively assessed volume and location (frontal, parietal and temporal) of white matter injury, measured from MRIs, were predictive of motor outcome, while only frontal injury was predictive of cognition in a large group of preterm infants (66).

Diffusion MRI quantitative measures have also been related to later behavioral development in infants at risk for brain injury. Using automatic voxelwise analyses of DTI showed that WM microstructure in full-term newborns correlates with neurodevelopmental outcome at 2-years (67). Another study on neonatal connectome (detected using deep learning approaches) at birth showed its predictive value on the 2-years cognitive outcome in both full-term and preterm infants (68), with connections involving the frontal lobe being the most important for classification. Smyser et al. (69), using a multivariate pattern analysis on resting-state functional MRIs from preterm infants compared to term controls, were able to estimate birth gestational age, and thus, brain maturity, with an accuracy of 84%.

Therefore, MRI advanced techniques provide direct information on brain morphology, structural brain connectivity, microstructural integrity of both gray and white matter, and also on cerebral function (47), giving indirect insights into molecular and cellular impairment in relation to brain injury. Thus, ML application to neonatal MRI, combined with other clinical, behavioral, and electrophysiological (see next paragraph) markers, can play an essential role in early diagnosis and prediction of neonatal brain injury and long-term impairment.

## Cranial Ultrasound (CUS)

Ultrasound is a neonatal neuroimaging technique with several advantages over other neuroimaging techniques: it is considered less burdensome to the patient, requires no transport (e.g., to the MRI unit), or sedation, it can be performed at the bedside with acceptable disturbance to the infant. It can be initiated directly after birth and repeated if necessary (70). Ultrasound is seen as complementary to MRI because it still lacks several important neuroimaging features such as quantitative tissue analysis US. Furthermore, CUS is operator dependent, has a limited field of view, and variability across the quality of ultrasound machines. However, ultrasound technology developments are rapid, and ultrasound techniques such as elastography, ultrafast Doppler, contrast-enhanced ultrasound, and functional ultrasound are examples of techniques finding their way in routine neonatal care (71–75). Early and serial neuroimaging can provide valuable information about the timing and evolution of neonatal brain lesions in (pre-)term infants and enables visualization of (a-)typical brain maturation (76).

Trained ultrasonographers, using modern ultrasound systems, can detect most neonatal hemorrhagic and ischemic brain lesions and major congenital as well as maturational anomalies (77). The use of different and higher frequency transducers (allowing submillimeter resolution) and additional acoustic windows (e.g., the mastoid fontanel) improved visualization, resulting in a more reliable detection of abnormalities (78). Doppler sonography of neonatal brain vessels enables the evaluation of intracranial blood flow velocities and the patency of both arteries and veins (e.g., to diagnose sinovenous thrombosis, arterial vessel occlusions) (78). Modern ultrasound machines have advanced Doppler modes, allowing visualization and quantification of low flows (1–2 cm/s) in small vessels (100–200 μm) (79).

### ML in Cerebral Ultrasound Techniques

Both 2D and 3D ultrasound measurements are useful to study (a-)typical fetal and neonatal brain growth (80). For example, using 3D ultrasound measurements, ventricle volumes can be calculated to evaluate PHVD (81, 82). Machine learning can be applied to classify fetal brain ultrasound images as normal or abnormal, to detect non-typical brain growth, and detect general and focal brain injury (e.g., IVH) on neonatal CUS (83). ML is very effective in ultrasound analysis by modeling complex multidimensional data (84).

Ultrasound elastography is a relatively new technique that calculates tissue stiffness and is used to study (ab-)normal neonatal brain development. Two types of elastography are frequently used in neonatal CUS studies: 1. strain elastography (using external compression) and 2. shear wave elastography (using applied acoustic energy). Contrast-enhanced neonatal CUS (CE-CUS) is another promising technique to study microvasculature and cerebral vascular autoregulation in infants at risk for brain injury (e.g., infants with HIE, infants with congenital heart defects) (85, 86). Contrast-enhanced neonatal CUS uses injection of gas-filled microbubbles to study blood flow. CE-CUS also allows targeted (localized) medication delivery, which has potential future use for localized drug delivery in the brain (87). Another fast-developing ultrasound technique that holds promise for neonatal care is ultrafast doppler (UFD). Perinatal brain injury is commonly associated with inadequate brain perfusion, and UFD can be used to study microperfusion in detail (72). Combining continuous UFD with EEG could unravel the relationship between cortical electrical activity and perfusion (e.g., infants with HIE and seizures) (71).

Because of the large amount of data that the above-discussed ultrasound techniques generate, the integration (registration) with other imaging- and neuromonitoring techniques, and the observer dependence, ML will play a significant role in the future of neonatal CUS. ML will be needed to design clinical decision support algorithms that take several individualized variables into account.

## The use of EEG/aEEG for Precision Medicine

Newborns with vital instability or at risk of serious morbidity are admitted or transferred to the NICU, where vital parameters such as heart rate, blood pressure, oxygen saturation, and other measures are closely monitored. Additionally, brain function monitoring is essential. EEG can monitor brain function, giving continuous, long-time, and high-resolution data on cortical function. Thus, EEG is a crucial tool for precision medicine and a tailored approach to neonatal brain injury. EEG can be useful for precise diagnosis, evaluation of treatment efficacy, and prognosis. However, interpreting conventional EEG presents significant challenges to clinicians, and the most prominent current limitation is the need for expertise in the interpretation of EEG traces (88, 89). Thus, most NICU currently use the filtered and time-compressed EEG trace (aEEG). aEEG is a non-invasive, inexpensive, bedside tool that evaluates the brain functional status of the newborn, with a relatively easier interpretation based on background patterns recognition. This technique is a powerful tool for the prediction of neurodevelopmental outcome in both preterm and term neonates.

In the last decade, the automatic classification of EEG/aEEG has been developed (90). Different automatic algorithm classifications of background patterns, sleep-wake cycling, and seizure detection have been investigated using machine learning approaches (90). This paves the way for future incorporation of these algorithms in the daily neuromonitoring of newborns at risk. The first positive results were obtained to predict adverse seizure-related outcomes in critically ill children, albeit in a small number of patients (91). Recently, an ML algorithm for neonatal seizure recognition, ANSeR, was investigated in a randomised controlled trial and was found to be safe and able to detect neonatal seizures. However, it did not yet improve the identification of individual neonates with seizures (38).

### EEG/aEEG in HIE and Stroke Patients

Term infants with NE need continuous monitoring of brain function using aEEG/EEG. The visual interpretation of the background pattern is a useful tool to monitor the recovery of cortical activity after HI injury (92, 93). Mainly, the normalization of the EEG after HI injury correlates with the outcome at 2 years of age. This process goes through different recovery steps from almost no electrical activity at the time of injury to the increasing number of bursts, toward a more continuous EEG with the appearance of sleep-wake stages (92, 94–96). For more personalized, brain-oriented care, visual EEG interpretation requires high expertise, and the evaluation incorporates multiple EEG characteristics such as continuity, amplitude, frequency, symmetry and synchrony, presence of sleep stages, and clinical information regarding gestational and postnatal age, differential diagnosis, administration of sedatives (94). This high expertise is not always continuously available in the NICU. Thus, the development of real-time, automated EEG analysis algorithms could be very valuable to assess cortical brain activity for clinical management, treatment evaluation, and prognosis. An important attempt was performed by Stevenson et al. (94) who developed a method for automatically grading the degree of EEG abnormality in neonates with HIE. EEG signals were post-processed based on EEG automated classification of abnormalities and assigned to one of four long-term EEG grades, resulting in 83% of EEG correct grading from 54 neonates. Lofhede et al. (90) managed to achieve 100% correct classification when separating burst suppression EEG from all other EEG patterns and 93% true positive classification when separating quiet sleep from the sleep stages in term infants. Burst suppression (BS) has also been associated with poor outcome (97), allowing for the analysis of interburst intervals (IBI) to be used as a feature that can assess the recovery of the infant's brain. New machines for cerebral functional monitoring incorporate automatic and real-time IBI calculation algorithm (IBI%), making it available for the daily clinical management of these infants.

Furthermore, as already stated, the presence of seizures can be an indicator for neurodevelopmental outcome, as they can be caused by HIE (98) or perinatal stroke and can be detected through EEG data or clinical observation (92, 99–102). Automated seizure detection algorithms (SDA) are being developed with a reasonable performance compared to human expertise (103, 104) and with the advantage of being more objective, capable of analyzing long EEG recordings with low false detection rates and low missed seizures rates (105). Yet, currently available SDAs show significant limitations since seizures can be of short duration, low amplitude, and possibly migrate from channel to channel, with large intra and interpatient variability of seizure morphology and repetitive patterns (106). Furthermore, there is a high number of artifacts both of biological or technical origin mimicking seizures, that in combination with the low incidence of seizures and the wide range of normal rhythmic background activity (varying across gestational ages and post-natal ages) can reduce the power in seizure detection rates and increase the number of false-positive detections (107).

Analysis of the newborn's sleep-wake cycle (SWC) can also provide helpful insights on outcome in infants with brain injury (108). Regular SWC can distinguish those with proper brain integrity from those with HIE (109), both in full-term (96) and extremely preterms (110). Recent work suggested that decreased EEG delta-frequency power and longer periods of quiet sleep, and lower sleep-wake state entropy were also predictive of worse neonatal neurobehavioral scores (110). Despite many publications regarding ML and the development of automatic EEG/aEEG algorithms, there are still studies failing to establish the long-term predictive value of early aEEG/EEG characteristics in neonates (111).

A possible solution to allow a more comprehensive picture of the brain and thus yield more consistent, personalized, and reliable results should be the use of a combination of different measurements of brain dynamics, such as aEEG/EEG, NIRS, and MRI, together with the clinical neurologic examination. Nowadays, only a few studies on newborns have focused on the use of combined early aEEG/EEG and other cerebral monitoring techniques for the prediction of future outcome, with some promising results (112–116).

### EEG/aEEG Concerning IVH-PVHI

Preterm infants are at risk for peri/intra-ventricular hemorrhage, especially during the transition phase, with associated adverse outcomes such as death or neurodevelopmental delay (117). Cerebral functional monitoring is essential to monitor preterm brain function during the first postnatal days. aEEG/EEG is the only effective bedside tool available in the NICU (118, 119).

In extremely preterm infants, EEG/aEEG develops through to full term age showing increasing continuity of the background patterns, appearance of specific transient waveforms typical of prematurity, and the appearance of sleep-wake cycling (120). Assessing the infants' EEG recording can give insights into individual brain maturation in relation to GA and postnatal age (PNA), and serial recordings can help determine the timing and severity of brain injury and, thus, outcome prognosis in this high-risk group (120). Therefore, cerebral functional monitoring (CFM) using aEEG/EEG is critical for diagnosis, prognosis, and treatment in the newborn period (120). ML approaches have been used to analyze several clinical factors in 230 very preterm infants to predict the risk of intracerebral hemorrhage with good predictive ability achieved with different combinations of clinical and laboratory parameters (121). However, the developed models need to be tested further in new larger datasets before being used in the clinics.

**Figure 2:**
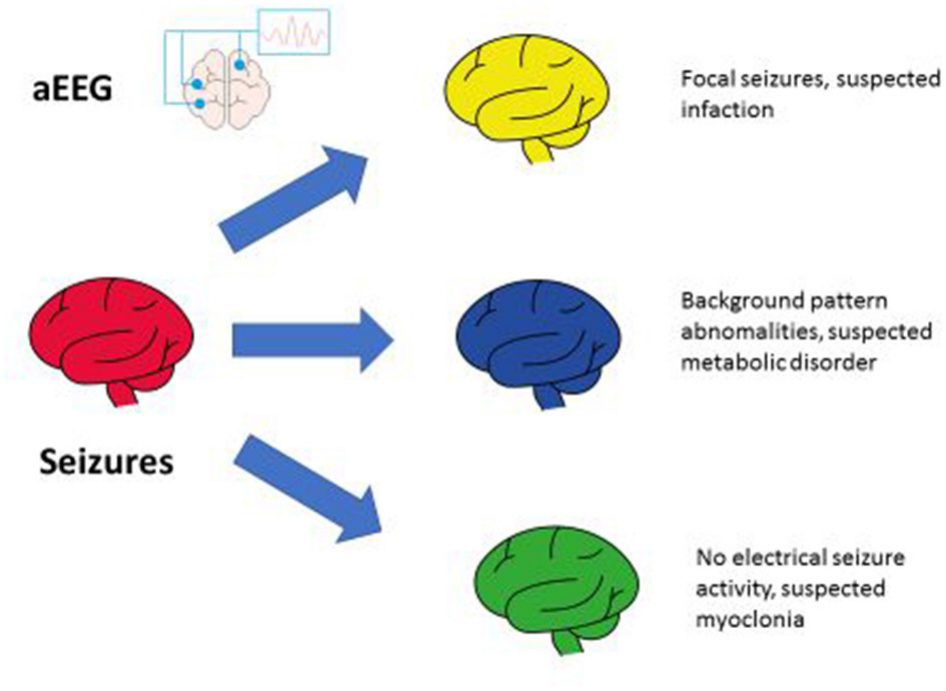
aEEG for neonatal seizures.

Regarding neonatal seizures in preterm infants, these are a distinctive sign of neurological dysfunction in early life, and diagnosis is always challenging in this group. Clinical features, when present, can often provide valuable clues about etiology. However, the majority of neonatal seizures are subclinical. Conventional video EEG and aEEG represents the gold standard for diagnosis, but ~15% of patients will require more sophisticated algorithms for diagnosis, including metabolic and genetic screening (110, 122, 123) (Figure 2). Currently, the standard recommendation is to monitor all neonates at high risk for seizures with long-term video-EEG (124) and to develop brain-oriented NICUs where neonatologists and pediatric neurologists would collaborate for early diagnosis and *ad hoc* treatments based on electroclinical phenotypes and etiology (125). Further steps should be taken in this direction in a multicenter/multicultural approach.

## Near Infrared Spectroscopy (NIRS)

Near-infrared spectroscopy (NIRS) is a bedside technique that can provide valuable continuous information on neonatal cerebral blood flow, cerebral blood volume, and oxygen consumption (126–130). NIRS monitoring can help evaluate the balance between tissue oxygen delivery and consumption, allowing assessment of brain perfusion in critically-ill infants. Several studies have shown correlations between cerebral NIRS data and different neonatal conditions (e.g., anemia, hypotension, patent ductus arteriosus, hypoxia, hypocarbia, sepsis, HIE, stroke). The SafeBoosC studies have examined steering treatment on guideline-driven cerebral rSO_2_ monitoring in extremely premature infants to improve clinical outcomes (131). Cerebral NIRS monitoring has now become a useful addition to other monitoring tools in several neonatology departments. NIRS monitoring has been incorporated in several multimodal neuromonitoring approaches to assess neonatal cerebral functioning in the past decade (132). For example, NIRS is used to study cerebral vascular autoregulation, in which machine learning is applied to unravel the complex interactions between blood pressure, NIRS, and EEG data (114, 133–135). ML has several uses in NIRS data analysis such as artifact detection and correction, the quantitative evaluation of deep and shallow tissue layers, to analyze the high-frequency raw NIRS data signals to study in beat-to-beat variations within the NIRS signals and to cope with the large amount of data when multiple NIRS optodes are applied to the neonatal head (136, 137). We believe that with the help of ML, NIRS will be part of the multimodal neuromonitoring of infants at risk for brain injury to diagnose injury and steer treatment to prevent further injury and optimize neurodevelopment.

## General Movements Assessment (GMs)

Assessment of general movements (GMs) is a neurodevelopmental biomarker and evaluates the presence and quality of spontaneous movements originating in the brainstem (88). GMs begin in fetal life and are useful to build neural connectivity between motor and sensory systems. The presence of specific movement patterns such as cramped-synchronized at term equivalent age, together with the absence of fidgety movements at 3–5 months, are predictive of the development of cerebral palsy and other developmental problems (138). GMs has a sensitivity of 98% for cerebral palsy (CP) prediction and represent, together with neonatal MRI (86–89% sensitivity) and the HINE (90% sensitivity), the best predictive tool for detecting cerebral palsy before 5 months' of age and as early as by 3 months (139–142). Limitations to the broader use of GMs evaluation is the lack of trained clinicians and its subjective nature. Recently, an attempt to a more objective and cost-effective alternative based on the automatic video-based assessment of GMs has been made (143–145). In the paper by Orlandi et al. (144) retrospective videos were evaluated using automatic analysis, and GMs were classified as typical or atypical using different classification algorithms. This retrospective study showed up to 92% accuracy in predicting CP. More effort should be made in this direction to support clinicians in early diagnosis and treatment.

## Hammersmith Infant Neurological Examination (HINE)

As previously mentioned, HINE can predict the development of cerebral palsy before 5 months of age with 90% sensitivity. There is evidence that the congruent combination of abnormal GMs trajectory, abnormal MRI and low HINE score is even more accurate than the individual technique alone (139, 140). The use of HINE plays a role also in the determination of severity of disability, a very important matter for parents or caregivers. Severity of motor outcome is difficult to predict before 2 years of age due to the rapid brain growth and re-organization in response to external stimuli and therapy. Thus, developing of motor skills but also the inconstant and changing presence of hypertonia (140), outcome prediction should always be discussed cautiously and based on standardized examinations. In particular, the following HINE cutoff scores predict the probable severity of motor outcome before 2 years of age: 50–73 Indicates likely unilateral CP (i.e., 95–99% will walk), ● <50 indicates likely bilateral CP. Furthermore, a score <40 at 3–6 months indicates the high chance of walking inability (142).

## Large Databases

Many preterm infants are affected by the same neonatal neurological incidents. However, their internal variation in inflammatory response, environmental expositions, and (epi)genetics can influence the etiology and treatment response. Identification of individual risk factors and pathophysiological reactions can lead to targeted interventions. The use of electronic health records (EHR) has created vast amounts of clinical data on infant treatment. Utilizing the knowledge extracted from this data has the potential of providing individualized treatment plans (36). Large databases consist of a combination of individuals. These databases provide an insight into the epidemiology of neonatal disease with trends over time and the distribution of risk factors (146). Neonatal research networks, such as the NICHD and Vermont Oxford network, are collaborative networks that combine data from different hospitals. They can combine relevant information on relatively rare diseases affecting the newborn infant using large numbers. Shankaran et al. (147) described a cohort of 4,216 infants to assess post-hemorrhagic dilatation outcomes in extremely preterm infants. They were able to identify several predictors of neurodevelopmental impairment or death, such as surgery for retinopathy of prematurity, even though the incidence in a general NICU would only be a few cases.

Even more significant numbers can be obtained using nationwide databases. Most of this database research is limited to general mortality and morbidity trends for extreme preterm birth (43). Matsushita et al. (148) used the Neonatal Network of Japan database to identify risk factors for epilepsy at 3 years of age in VLBW infants. As only 1.7% of the cohort developed epilepsy, it would have been almost impossible to identify clinically relevant risk factors in a smaller cohort. Technological innovations make it possible to combine different information sources and provide information on a more detailed level. Clinically collected data from EHR with well-regulated, international, and privacy proof unrestricted access for researchers, such as the MIMIC-III and AmsterdamUMCdb database, and the increased availability of raw trial data could bring about a revolution in research on preterm neonates (149–152).

## (Epi)Genetics and Omics—Future

The future concept of personalized medicine will be based on the idea that by using individual genetic/metabolic information, scientists may ensure the most appropriate treatments to the right patients—thus, “the right drug, at the right dose for the right person” (153). Genomics and epigenetics, i.e., the interaction between the genome and the environment, are changing the concept of clinical medicine, and this is particularly true in the field of neonatal neurology. Neonatologists and pediatricians have the unique chance to ensure that young patients derive maximal benefit from these new technologies.

In a recent study, epigenetic changes measured in blood leucocytes and analyzed using AI/ML techniques appeared to predict cerebral palsy accurately and provide crucial information on the pathogenesis of long-term disability (12).

Both genomics and epigenetics will provide clinicians new insights into the biological basis of health and disease (154). This will also lead to the sometimes-challenging choices of both the clinicians and the patients/families. Furthermore, understanding the mechanisms through which the environment exerts changes on genome expression will give new possibilities for new treatments by modulating gene expression and should be further investigated (155). In a not far-off future, knowledge of patients' genomes will help improving diagnosis and, through informed prediction of individual drug metabolism and responsiveness, the individualized selection of therapies.

Metabolomics can also provide valuable information for outcome prediction. Metabolites offer a unique signature potentially usable to predict neonatal diseases and evaluate disease progression and treatments' effect (156–158).

Metabolomic analysis performed in cord blood predicted the development of NE with an AUC of 0.67, with lactic acid and alanine as primary metabolite predictors for NE. When metabolomic analysis results were combined with clinical data, the AUC rose to 0.96 (13). Moreover, urinary metabolic spectra of extremely preterm infants early after birth were associated with moderately to severely abnormal cortical grey matter and white matter abnormalities at MRI performed at term equivalent age MRI (158). A growing number of studies had been published on this subject since metabolomic has the advantage of being rapid and non-invasive. Thus, metabolomics could be useful for monitoring early cellular injuries and cell death during perinatal insults. Therefore, it can pave the way for the early preventive measure to improve the neurodevelopmental outcome of the affected newborns.

## Conclusion

The machine learning approach will provide more detailed information using AI for MRI, CUS, EEG, NIRS, and GM/HINE. An algorithm combining all techniques might give the best decision support tool for defining risk factors for brain injury or impaired brain development, therefore enabling better treatment and long-term outcome. In IVH, PAIS, and brain injury after HIE, MRI can improve personalized prognosis and treatment plan. EEG and aEEG provide more information on the brain than just a background pattern and seizure activity. Together with the HINE, GMs are the best predictive tools for early detecting cerebral palsy, and automatization of GMs classification can increase the rate of early diagnosis.

However, for the utilization of this potential, more expertise and the dissemination of knowledge is essential. Machine learning has the prospective of alleviating the task of bringing all the pieces of knowledge together. With the increasing amount of data on the infant in the NICU, it will become nearly impossible to interpret all these variables for a clinician and use it for the benefit of the individual patient. Artificial intelligence can fill (part of) the gap of knowledge and interpretation. Especially when genetics, epigenetics, biomarker research, and metabolomics will provide us with even more variables in the near future. As the era of AI, -ethics, and –omics is approaching, we must consider the Ethics. Will all babies benefit equally from precision medicine? Most efforts to personalised medicine require a high resource setting. MRI, continuous monitoring with ML interpretation, whole-genome sequencing, and even fully equipped NICU are not available in most parts of the world. Furthermore, to make an individual treatment plan and risk assessment, many assumptions are taken into account. They hold the risk of bias and even discrimination. Attempts to personalise treatment plans must include careful ethical consideration; therefore, it should eventually be considered a decision support tool. Careful monitoring of infants in the perinatal period can potentially identify and improve neonatal brain injury treatment. “Precision medicine toward personalised care” is the aim for the near future.

## Author Contributions

MT, DV, and MB: conception/design, literature review, manuscript draft and review, critical review, and approval of final manuscript. JD: literature review, manuscript draft and review, critical review, and approval of final manuscript. All authors contributed to the article and approved the submitted version.

## Conflict of Interest

The authors declare that the research was conducted in the absence of any commercial or financial relationships that could be construed as a potential conflict of interest.
